# How a token-based game may elicit the reward prediction error and increase engagement of students in elementary school. A pilot study

**DOI:** 10.3389/fpsyg.2023.1077406

**Published:** 2023-04-05

**Authors:** Marcus Eckert, Viviane Scherenberg, Clemens Klinke

**Affiliations:** ^1^Department of Psychology and Pedagogy, Apollon University of Applied Sciences, Bremen, Germany; ^2^Department of Public Health and Environmental Health, Apollon University of Applied Sciences, Bremen, Germany

**Keywords:** token-based reinforcement, classroom management game, reward prediction error, elementary school, gamification

## Abstract

Student engagement is essential to academic success and student-wellbeing. In the past, fostering engagement though extrinsic rewards has often been found to be of limited effectiveness over the long term. However, extrinsic rewards are important for improving engagement with non-intrinsically rewarding activities. Thus, in the present study a mechanism that is meant to prolong the effects of extrinsic rewards was investigated: the reward prediction error. This error occurs when rewards are awarded contrary to the awardee’s expectations. In a quasi-experiment, 39 elementary school students participated in a classroom-based game, which was supposed to motivate them to solve math exercises. It combined reinforcement with elements of luck, which were supposed to elicit the reward prediction error. After 2 weeks, the intervention group had completed significantly more math exercises compared to a pretest and, importantly, also more correctly solved exercises than a control group. This suggests that game-based reinforcement that elicits the reward prediction error might help to increase student engagement over the medium term. It furthermore highlights the importance of applying gamification elements not only digitally but also in analog settings.

## Introduction

Student engagement is associated with academic success and seems to foster wellbeing in school (Upadyaya and Salmela-Aro, 2013; Lu et al., 2022). In past research it has been defined by the effort that is put in activities that are associated with positive learning outcomes (Krause and Coates, 2008). Other research extended this definition to also include institutional factors (Kuh, 2009), yet most studies discuss it in the context of individual student behavior (Trowler, 2010). An explanation for the positive influence that engagement has on academic performance might be that students’ classroom engagement increases their motivation (Reeve and Lee, 2014) and vice versa. Motivation is also important for students’ learning progress and initiates and sustains goal-directed activity (Pintrich and Schunk, 2002; Valerio, 2012). Thus, in classroom management, methods should be applied that increase both classroom engagement and classroom motivation.

Student engagement is influenced by a variety of factors. Intrinsic motivation, social support, self-efficacy and perceived autonomy predict engagement (Miller and Wallis, 2009; Lu et al., 2022). The self-determination theory (SDT) proposes three factors that foster student engagement and motivation: The need for competence, relatedness, and autonomy (Ryan and Deci, 2020). Previous research furthermore lines out that certain classroom management strategies that build on these factors improve motivational outcomes in elementary education (Korpershoek et al., 2016). If classroom management strategies induce intrinsic motivation students will be more engaged, work harder and longer on their assigned tasks (Miller and Wallis, 2009).

In contrast to intrinsic motivation, SDT maintains, that extrinsic motivation cannot lead to effective learning. However, Luria et al. (2021) challenge this assumption with an alternative approach, considering insights from the neurobiology of memory. They argue that seeking and reaping rewards activates dopamine-related mechanisms which influence behavior, procedural learning, and in line with recent research, also the formation of declarative memories. Thus, extrinsic rewards may foster engagement and learning in the classroom as well. Luria et al. (2021) suggest that gamification may promote learning – and it could fulfill the needs addressed by the SDT and extrinsic rewards. Gamification elements can be used as classroom management strategies (Morano et al., 2021) in order to foster motivation, divergent thinking, creative tendency, the relationship among students, autonomy, and increased performance (Cunha et al., 2018; Xi and Hamari, 2019; Chen et al., 2020). Furthermore, these effects seem to be independent of age (Putz et al., 2020). In order to use extrinsic motivational elements in gamification, one possibility is the integration of token economies (Homer et al., 2018).

Especially in elementary school classrooms, it seems to be common to use token economy systems (Doll et al., 2013). There is a body of evidence that material reinforcement influences elementary school student’s behavior (Igbo et al., 2016). Compared with baseline and response cost, findings indicate that token economies were most effective in increasing academic engagement and reducing inappropriate classroom behavior (DeJager et al., 2020). But until today, there is a lack of methodically solid research, that is in line with What Works Clearinghouse (WWC) criteria, supporting evidence of token economies as best practice in the classroom (Maggin et al., 2011). WWC criteria define evidence for educational programs and interventions.

In applying token systems, teachers should be aware of different reinforcement schedules: To initiate new target behavior, continuous reinforcement should be applied, whereas variable rate schedules tend to lead to more stable behavior change (Worsdell et al., 2000; Troussas et al., 2017). Regarding the neuronal underpins of reward, dopamine neurons of the ventral tegmental area (VTA) and substantia nigra send information about rewarding events to brain structures involved in motivation and goal-directed behavior (Schultz et al., 1997; Palminteri and Pessiglione, 2017; McCane et al., 2021). It is important to understand that reinforcement does not activate the dopamine neurons *per se*. Schultz et al. (1997) found that the presentation of unexpected rewards activated dopamine neurons, whereas repeated and predictable presentation of rewards decreased activity of these neurons. In a nutshell, if rewards are predicted, dopamine neurons do not fire, however a reward which exceeds expectations, activates dopamine neurons. This is called the reward prediction error (Schultz, 1998, 2017; Lerner et al., 2021).

Dopamine promotes synaptic modifications, which are associated with learning and changes in behavioral habits (Glimcher, 2011). Thus, the reward prediction error could underlie reinforcement learning mechanisms, however only when the reinforcement is unpredictable (Schultz, 2016). With regard to operant schedules, it should be noted that in a continuous reinforcement schedule, the reward followed each exposure of the target behavior, whereas in the intermittent and variable ratio reinforcement schedule the reward follows not predictable to target behavior. Accordingly, Hogarth and Villeval (2014) found that intermittent reinforcement leads to higher mean performance and to more persistence in efforts of individuals compared to continuous reinforcement. In addition to this, previous research highlighted that variable ratio schedules suit the classroom setting well (Lee and Belfiore, 1997; Ivy et al., 2017; Rizk et al., 2022). Thus, the persistence of classroom engagement should be higher if it is reinforced through variable ratio schedules.

Moreover, beyond motivational and behavioral effects, recent research identified some more benefits of the reward prediction error, which could be relevant for classroom engagement and academic success. In a brief literature review Ergo et al. (2020) highlighted that the reward prediction error may modulate declarative learning, because dopamine influences (declarative) memory traces in the brain. Miller and Wallis (2009) pointed out, that the “reward prediction error signal is ideal for instructing when and what the system should learn and consequently enables the organism to acquire the representations necessary to achieve reward” (p. 104). It is suggested that dopamine related reinforcement influences the working memory and executive control functions of the prefrontal cortex, since “the mesencephalic dopamine system projects throughout the basal ganglia and frontal cortex” (Holroyd and Coles, 2002, p. 697). In addition to this, the reward prediction error is associated to enjoyment, especially in gambling tasks (Rutledge et al., 2014).

The reward prediction error might also explain the effectiveness of game elements both in increasing dopamine levels and improving performance. In game studies, brains of gamers showed an increased dopamine level during play compared to controls (Mayo, 2007; Murad, 2017). Consequently, Mayo (2007) found that performance of the players was associated to the amount of dopamine that was released in the participant’s brains. For a computer-based gamification of a training for children with developmental dyscalculia, Kucian et al. (2011) found that the gaming-aspects of the training increased engagement with the training exercises. In line with Gruber et al. (2014), not the value of the reward but the unexpectedness and the uncertainty in games play a critical role. Additionally, unexpected rewards generate curiosity and focus the attention that is needed to learn (Nabar et al., 2018).

In line with Luria et al. (2021), we assume, that gamification may promote learning, “regardless of whether its motivation base is intrinsic or extrinsic, but rather as a function of the goal on which it is centred” (p. 12). Accordingly, Petursdottir and Ragnarsdottir (2019) found that a token economy increases academic engagement. Thus, in the present study a gamified token economy that is supposed to elicit the reward prediction error by incorporating different reinforcement schedules will be investigated. Compared to continuous reinforcement, variable ratio reinforcement schedules may be more appropriate for developing reading or math computational fluency skills (Hulac et al., 2016). Moreover, it was found, that variable ratio reinforcement is more effective than continuous and fixed ratio reinforcement (Latham and Dossett, 1978; Houten and Nau, 1980). Variable ratio schedule reinforcement means that reinforcement follows a variable and unpredictable number of rewards, but the average number of rewards is fixed. For example, slot-machines use this schedule.

Previous research suggests that central dopaminergic substrates play a role in positive reinforcement (Ettenberg, 1989). Although continuous reinforcement quickly increases the probability of the target behavior (Worsdell et al., 2000), it is likely that the experience of reward will decrease with repeated reception of these token and the students’ dopamine levels will drop (Schultz et al., 1997). This may explain the benefits of variable ratio reinforcement since the reward cannot be predicted. Thus, regarding a token-based classroom game, a complex game mechanic is needed to elicit the reward prediction error.

While in the beginning of a token-based game the continuous reception of the token (continuous reinforcement) may be experienced as rewarding, it is likely that the experience of reward would decrease with repeated reception of these token (in our case cards) (Schultz et al., 1997). Thus, if procedures of exchanging token from lower into higher level-token include random chance, these procedures could be seen as a variable ratio schedule of reinforcement and may elicit the reward prediction error. Moreover, this principal is typical for many games: from slot-machines (Hurlburt et al., 1980) to serious gaming. Nagle et al. (2014) highlighted “the importance of in-game rewards, especially […] a variable-ratio schedule with a player-centred design was able to elicit high levels of enjoyment and performance from participants, and therefore could be a viable reward scheduling method in serious games.” (p. 44). Nagle et al. (2014) identified a large increase of the game score as an in-game rewards used in serious games. An equivalent for large increases in game scores that is typical for many games are exchange-procedures, which define rules how token of a low level could be changed into token of a higher level following a variable ratio schedule.

If game-based token systems elicit a reward prediction error, they should promote classroom engagement, cognitive achievement, and wellbeing. The purpose of this study is to investigate whether a game-based token system, which considers the conditions of the reward prediction error, increases the classroom engagement of elementary school students. Regarding elementary school and gamification, most studies investigated digital gamification in the past (Indriasari et al., 2020; Nadi-Ravandi and Batooli, 2022). But to the best of our knowledge no study ever investigated a token-based game in elementary school on classroom engagement in a non-digital setting. Thus, we hypothesize that a non-digital game which accompanies the math lessons in a third-grade class in elementary school will increase classroom engagement as well as achievement in math exercises. To lay the foundation for future randomized controlled trials that require considerable human resources from the schools and might be a burden to their organizational structures, a quasi-experimental study with a small sample sized was planned. For this purpose, we developed a token-based game which is tested in this pilot-study, for the first time.

## Methods

### Participants and procedures

The participants of this study were 77 third-degree students at an elementary school (female *n* = 35). Since all students who agreed to participate, were included in this pilot study, they represented a convenience sample. The inclusion criteria were that all third classes followed the same curriculum and worked on the same math exercises provided by the course literature. All Students completed a pre- and a post-measure. In the pre-measure, we included 83 third-class students attending four different classes in one, while 77 third-degree students (female *n* = 35) completed the post-measure (8% dropout). The mean age was 8.6 years (SD = 0.61). For this quasi-experimental study design, two classes were randomly assigned to the intervention group (IG) and two classes to the control group (CG). In the IG were 39 students (*n* = 18 female), whereas 38 students were in the CG (*n* = 17 female).

All classes did the same math exercises concerning basic arithmetic (e.g., 456–237 =?). The program ran about 2 weeks, all classes had four lessons of math per week. In the classes assigned to the IG the exercises were gamified by the token-based game “Team of Animals” which is described below. The game in the IG was executed by the teachers. The CG got exercises as usual.

In one lesson before the token-based training and one lesson after the token-based training, pre-and post-measures were conducted. All procedures are in line with the ethical guidelines of the ethical committee of the APOLLON University of Applied Science in Bremen.

### Measure

In order to measure engagement in math, from a pool of math-exercises, 65 exercises were randomly chosen for each measurement (pre and post). Engagement on an individual level was operationalized similarly in earlier research by for example monitoring the submission of assignments or class attendance (Domínguez et al., 2013; O'Donovan et al., 2013). This operationalization needs to be distinguished from other approaches that measured engagement through surveys that assessed different facets of engagement (e. g. active learning or participation in academic activities; Coates, 2007, 2009). The math exercises were derived from additional material of the course literature that was used in all classes. Thus, we ensured that the students were able to solve the exercises. The teachers furthermore evaluated the suitability of the exercises for this study. The tests were conducted by the investigator. The students received the instruction to solve as many exercises as possible within 10 min. Additionally, they were informed, that they would not get any grade. To test general engagement, the amount of completed exercises were counted as the behavioral outcome measure. Since it is unclear, whether students disregard accuracy in order to complete more exercises, for an additional analysis only correctly solved exercises were counted. Doing so, for each student two values were identified at each timepoint: the amount of (1) completed and (2) correctly solved exercises.

### Intervention: Game “team of animals”

The game “Team of Animals” is a token-based game, which can be played for the duration of one up to 3 weeks. For reasons of feasibility and in order to booster engagement (see below), the students are divided into four groups. For this pilot-study, a duration of 2 weeks was defined.

**Figure 1:**
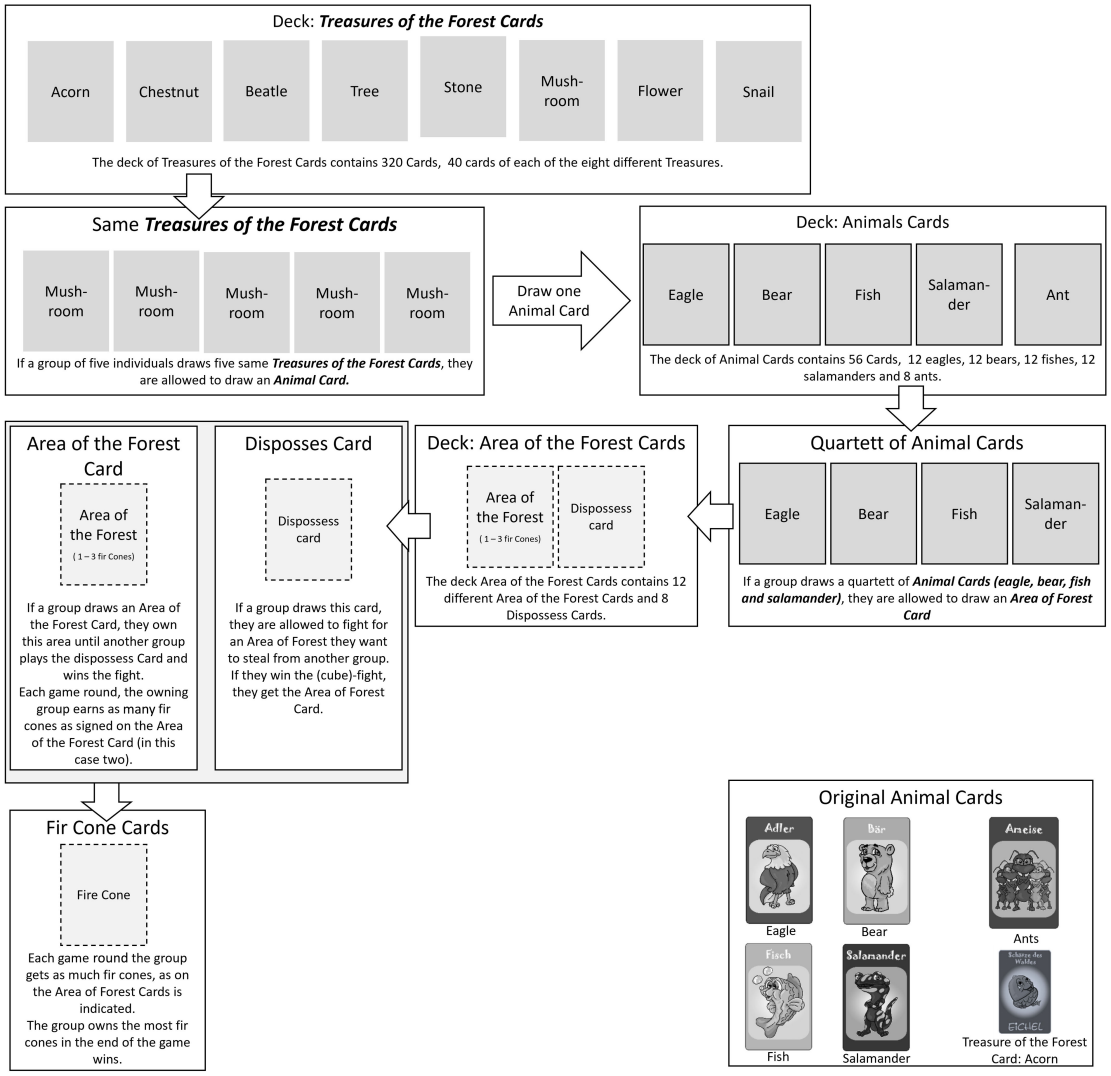
Mechanics of the game team of animals for original cards.

In the game, the target behavior is reinforced by receiving cards called “Treasures of the Forest” (TotF). These are the basic token of the game (low level token), as the students receive the cards for solving exercises in the math training lessons continuously. The game “Team of Animals” uses elements of random chance to elicit the reward-prediction-error and to keep the students’ dopamine levels high. The combination of reinforcement and random chance is realized by a game mechanic involving rules for exchanging the cards (lower-level token) into cards of higher order (e. g. treasures-of the-forest-cards to animal cards, see Figure 1).

By drawing a “Treasure-of-the-forest-card” the players get one of eight possible treasures as motives (e.g., a tree, a mushroom, a flower etc.) randomly. If one group collected *n* treasure-of-the-forest-cards with the same treasure-motive, they are allowed to exchange these cards into one animal-card (exchange into higher level token), which they also draw randomly. The number of necessary treasures of the forest cards *(n)* is defined by the amount of group members. For example, if five players are in one group, they must collect five cards of the same treasure-motive (e.g., a tree), in order to exchange them for one animal card.

In the deck of animal-cards are four animals (fish, bear, eagle and salamander). The students draw one of these animals at random. Altogether, the deck includes 56 animal-cards. If a group has one quartet of animals (i.e., each of the four animals at least once), they are allowed to draw one area-of-forest-card. Since this is driven by random chance, this game mechanic is designed to elicit the reward prediction error.

In the area-of-forest card deck there are 12 area-of-forest-cards and six dispossess-cards. By owning area-of-the forest cards, the groups eventually earn fir-cone-cards. On the area-of-forest-cards, a number indicates how many fir-cone-cards the group receives each game round. A game round is defined as one math lesson. In the end of each lesson the students earn fir-cone-cards which lead to another rewarding experience.

From a student perspective, the aim of this game is to collect as many fir-cone-cards as possible. The group that collected the most fir-cone-cards in the end, wins the game. Since the area-of-forest-cards are limited, the groups can dispossess each other’s area-of-forest-cards in a special dice-procedure. For this, they need a Dispossess-card which is part of the area-of-forest-cards-deck and hence drawn randomly. Once more, winning the dice-procedure by chance may elicit the reward prediction error. In summary, all procedures of exchanging cards from lower to higher levels are based on a variable ratio schedule of reinforcement and may elicit the reward prediction error.

In the IG, the students draw for every solved exercise one treasure-of-forest-card. For all other game-actions (e.g., changing cards), the students were granted approximately 5 min in the end of every math-lesson. Although these rules seem to be very complex, through trial and error the students understood them very fast.

## Results

We first tested whether all assumptions for an ANOVA were met, since the sample sizes were small. A non-significant box-test (*Box´ M* = 4,656, *p* > 0.05) indicated equality of co-variances. Regarding engagement, a Levine-test indicated that variances at post-measure seemed to be equal (*F*_post_ = 2.066; *p* > 0.05), however at pre-measure variances differed (*F*_pre_ = 12.655; *p* < 0.01). A similar pattern was found for correctness (*F*_pre_ = 6.081; *p* < 0.05; *F*_post_ = 1.029; *p* > 0.05). Furthermore, skewness and kurtosis were investigated. A normally distributed variable should score ≤ ±2 for skewness and ≤ ±3 for kurtosis (Kline, 2005). While skewness levels were acceptable for all variables (all ≤ ± 2) for the IG at baseline the kurtosis was too high (3.45; see Table 1). To account for the violation of the assumptions of the ANOVA, we instead conducted nonparametric analyses. Friedman tests were conducted to investigate overall differences between the groups, which were followed up with Wilcoxon signed rank tests to compare the effects for each group separately. Table 1 provides descriptive data for all variables.

**Table 1:** Descriptive Statistics (Mean, standard deviation, skewness, and kurtosis).

	Intervention group							Control group						
	*N*	Mean	SD	Skewness		Kurtosis		*N*	Mean	SD	Skewness		Kurtosis	
				M	SD	M	SD				M	SD	M	SD
Engagement_pre_	39	41.25	10.42	−1.67	0.38	3.45	0.74	38	48.55	14.64	−0.23	0.38	−1.16	0.75
Engagement_post_	39	54.58	15.13	−0.63	0.38	1.35	0.74	38	49.81	18.42	0.07	0.38	0.05	0.75
Correctness_pre_	39	36.95	10.63	−1.21	0.38	1.50	0.74	38	43.63	14.34	0.10	0.38	−1.01	0.75
Correctness_post_	39	48.54	16.12	−0.24	0.38	0.48	0.74	38	44.89	18.46	0.29	0.38	−0.39	0.75

**Figure 2:**
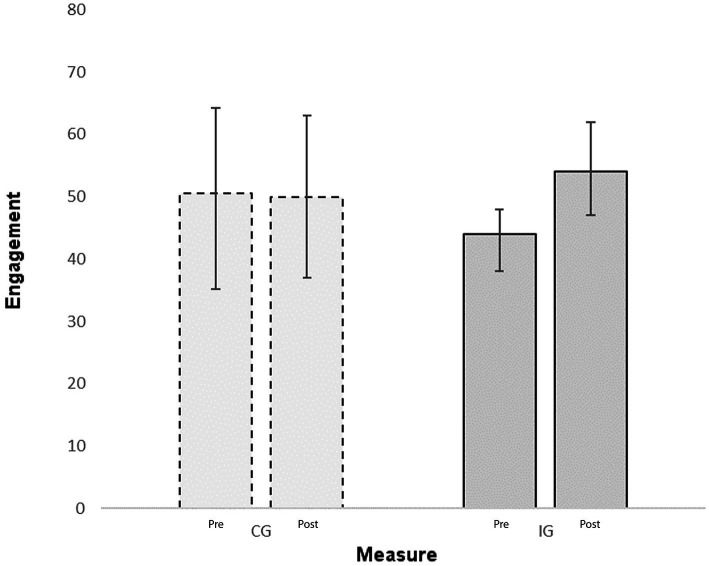
Engagement measured in solved exercises. The graph displays medians and interquartile ranges.

The Friedman test to investigate engagement included four variables: (1) Engagement in the IG at pre-measure, (2) engagement in the CG at pre-measure, (3) engagement in the IG at post-measure and (4) engagement in the CG at post-measure. Overall, the Friedman test indicated significant differences (*χ*^2^ = 27.59; *p* < 0.001). In a next step we tested pre-post differences with Wilcoxon tests. While no difference was found in the CG (Median_t1_ = 50.5; IQR_t1_ = 34.75–64.25; Median_t2_ = 50.0; IQR_t2_ = 37.00–63.00; *Z* = −0.392; *p* > 0.05), in the IG the pre-post difference was significant (Median_t1_ = 44.0; IQR_t1_ = 38.00–48.00; Median_t2_ = 54.0; IQR_t2_ = 47.00–62.00; *Z* = −5.223; *p* < 0.001). Figure 2 illustrates these effects.

The Friedman test that was computed to investigate differences in correctness also included four variables: (1) Correctness in the IG at pre-measure, (2) correctness in the CG at pre-measure, (3) correctness in the IG at post-measure and (4) correctness in the CG at post-measure. The Friedman test indicated significant differences overall (*χ*^2^ = 26.33; p < 0.001). Wilcoxon tests indicated no significant pre-post difference in the CG (Median_t1_ = 43.0; IQR_t1_ = 31.00–55.00; Median_t2_ = 42.0; IQR_t2_ = 30.00–59.00; *Z* = −0.754; *p* > 0.05), but in the IG a significant difference was found (Median_t1_ = 40.0; IQR_t1_ = 33.00–43.00; Median_t2_ = 52.0; IQR_t2_ = 36.00–59.00; *Z* = −4.97; *p* < 0.001). For an overview over all analyses please see Table 2.

**Table 2:** Overview over nonparametric analyses.

			Pre		Post					
		*N*	*Median_t1_*	*IQR_t1_*	*Median_t2_*	*IQR_t2_*	*Z*	*p*	*χ* ^2^	*p*
Engagement	IG	39	44.00	38.00–48.00	54.00	47.00–62.00	−5.22	0.000	27.59	0.000
	CG	38	50.50	34.75–64.25	50.00	37.00–63.00	−0.392	0.695		
Correctness	IG	39	40.00	33.00–43.00	52.00	36.00–59.00	−4.97	0.000	26.33	0.000
	CG	38	43.00	31.00–55.00	42.00	30.00–59.00	−0.75	0.451		

This table displays the medians and interquartile ranges (IQR) of the pre- and post-measures in IG and CG. Wilcoxon tests and related *p*-values indicate whether a change from pre- to post-measure in each condition is significant. The *χ*^2^-value and the related value of p indicate a significant overall effect for the two separate analyses yielded by the Friedman test.

## Discussion

In this pilot study we investigated whether a token-based game that should elicit a reward prediction error would increase the engagement in math exercises. We found that engagement in the IG was increased. Instead of impairing the correctness of the exercises by submitting more exercises, we found that accuracy also improved. Thus, the interventions may booster performance and engagement. Engagement in mathematics is essential for academic success (Morano et al., 2021).

Previous findings indicate that educational gamification increases student engagement, group work, activation, and commitment to learning tasks (Manzano-León et al., 2021; Mee et al., 2022; Nyahuye and Steyn, 2022). Segura-Robles et al. (2020) suggest that gamification increases satisfaction and enjoyment of the students which may increase the intrinsic motivation of students and was associated directly with a better performance. Regarding the present results, it seems likely that the increase of engagement was fostered by increased enjoyment while playing the game Team of Animals. Enjoyment is furthermore associated with the release of dopamine (Brooks, 2006). It is still not clear, whether these effects are partially mediated by the positive reward prediction error because we did not measure dopamine release in the striatum while playing the game. But considering the game mechanism, it seems very likely that this game elicits prediction errors. In line with previous findings, we assumed that a reward prediction error increases engagement and performance (Holroyd and Coles, 2002; Glimcher, 2011; Ottenheimer et al., 2020). Glimcher (2011) highlighted that reinforcement learning systems should use the reward prediction error signal. Accordingly, the present study indicates that this may succeed by administering a token-based game in the classroom. Since this study did not clarify whether the observed effect was mediated by the reward prediction error, future research is needed. Ergo et al. (2020) investigated whether the reward prediction error boosts declarative memory. It was shown that the reward prediction errors during declarative learning improved recognition if it was indicated whether the outcome is better or worse than expected (signed reward prediction error) compared to a so-called unsigned reward prediction error condition, in which it was merely indicated that the outcome differs from the expected outcome (De Loof et al., 2018). Thus, future studies could conduct the game “Team of animals” in a declarative learning setting. It could be signed when a reward is better than expectable, compared to an unsigned trial. If the recognition in the signed trial is better than in the non-signed trial, this would yield further evidence for the reward prediction error to mediate the effect. In line with this and similar to the present study, future research should conduct a three-armed trial, in order to investigate whether the reward prediction error mediates the effects of the game “Team of animals” on engagement and performance in math exercises. One arm should be a signed-, one arm a non-signed reward prediction error and one arm the CG. It seems likely that engagement and performance would be better in the signed arm, if the reward prediction error underpins the effects, found in the present study.

Furthermore, qualitative studies may investigate the subjective experience of the students playing the “Team of animals.” If students would be asked how the exchanging procedures in the game affected their subjective motivation, this could yield further evidence for the involvement of the reward prediction error. Since the present research is a pilot study, a replication with a larger sample size should consider this.

## Limitations

Several limitations need to be addressed. First, the present study had a small sample size. Moreover, the sample consisted of four classes. We aimed to test the game “Team of animals” in a small pilot study, in order to get a first estimate of the effects. In future studies larger sample sizes would be beneficial. Second, we tested engagement and performance with behavioral outcomes. But we did not record constructs like self-efficacy, emotion regulation or affect which may have influenced engagement and performance and may themselves be affected by the intervention. Future research should overcome this shortcoming. Third, no follow-up measures were conducted. Thus, we do not know anything about the stability of the effects. Regarding the stability of continuous and intermittent reinforcement, it could be interesting to investigate whether intermittent reinforcement indeed ensures greater stability of the effects. Thus, future studies should consider follow-up measures.

## Conclusion and implication

Beyond theoretical aspects, our results provide some practical implications. With regard to gamification in elementary school, a lot is known about digital learning games, but less about non-digital games. Our study yields preliminary evidence that a non-digital game seems to increase classroom engagement and performance in math exercises. Considering that the game “Team of animals” is group-based and incorporates a task oriented and cooperative climate into the experience of intergroup competition, effects on the social climate within the class should be measured. Moreover, the game may be applied to increase classroom cooperation and sportsmanship. It was shown that sportsmanship increases student conflict-resolution behaviors and decreases the frequency of student off-task behavior for elementary school students (Sharpe et al., 1995). Thus, effects on social behavior should also be investigated.

Another context in which the investigated mechanisms could be applied is flipped learning. Flipped learning is a learning mode that reverses the traditional in-class instruction and was found to increase student engagement even in elementary school (Hwang and Lai, 2013). A study of Pozo-Sánchez et al. (2020) found that gamification in the face-to-face phase of flipped learning increases motivation, interaction with teachers, and interactions between students in higher education. Regarding elementary schools, it could be valuable to investigate, whether gamification in the face-to-face phase of flipped learning has similar benefits, by implementing the game “Team of animals.”

## Data availability statement

The raw data supporting the conclusions of this article will be made available by the authors, without undue reservation.

## Ethics statement

The studies involving human participants were reviewed and approved by Gemeinsame Ethikkommission der APOLLON Hochschule (Bremen) und der HKS (Ottersberg). Written informed consent to participate in this study was provided by the participants’ legal guardian/next of kin.

## Author contributions

ME developing the intervention–planning and conducting the study–Statistics–writing. VS: co-writing. CK–supervising–co-writing. All authors contributed to the article and approved the submitted version.

## Conflict of interest

The authors declare that the research was conducted in the absence of any commercial or financial relationships that could be construed as a potential conflict of interest.

## Publisher’s note

All claims expressed in this article are solely those of the authors and do not necessarily represent those of their affiliated organizations, or those of the publisher, the editors and the reviewers. Any product that may be evaluated in this article, or claim that may be made by its manufacturer, is not guaranteed or endorsed by the publisher.
